# Ingrown Toe Nails

**Published:** 1897-10

**Authors:** 


### INGROWN TOE NAILS.

Dr. Andrews, in the N. Y. Medical Journal, recommends the following modification of Anger's method:

The toes and foot are first scrubbed with soap and water, followed by a solution of bichloride of mercury (1 to 1,000). For anæsthesia, ten to fifteen minims of a two per cent. solution of cocaine hydrochloride is sufficient if distributed in the following manner: Introduce the hypodermic needle from before backward, just under the part of the nail to be removed; as soon as the skin is pierced inject a drop or two and wait a moment before pushing it further; repeat the process until the root of the nail is reached. Withdraw the needle now, and beginning near the primary punctnre, distribute a few drops in the skin and soft parts along the outer side of the nail, nearly as far backward as the first joint.

Next wind a small rubber tube around the base of the toe. Before making an incision swab the whole granular area thoroughly with pure carbolic acid.

The first incision begins anteriorly somewhat beyond the edge of the nail, and is carried directly backward (separating a sufficient amount of the side of the latter) to a point about a fourth of an inch behind the true matrix. This is deepened through the soft parts, well down by the side of the phalanx.

The second incision begins anteriorly at the same point as the first, and curves outward and backward to join the posterior end of the primary incision. This is deepened through the soft parts around the outside of the nail until we reach the lowest part of the first incision.

Remove the wedge thus set free and search the posterior part of the wound carefully, so that no part of the corner of the nail or matrix shall be left.

If property fashioned, the flap which we have made will fit the opposite raw surface perfectly, and should be secured by one or two catgut sutures in front of the nail and the same number behind. Before removing the rubber band apply a moderately thick pad of gauze over the flap, and bind it tightly to the toe with a few turns of gauze bandage. Now remove the rubber tube and complete the dressing.

The patients walk home, and although there is often some pain the first night it does not last longer and they keep about with little discomfort. The dressing should not be changed in less than a week, unless there is some indication, and at the end of ten days or two weeks the cure will usually be complete.—*Charlotte Medical Journal.*

